# Subversion of the Immune Response by Human Pathogenic Mycoplasmas

**DOI:** 10.3389/fmicb.2019.01934

**Published:** 2019-08-21

**Authors:** Lianmei Qin, Yiwen Chen, Xiaoxing You

**Affiliations:** Institute of Pathogenic Biology, Hengyang Medical College, Hunan Provincial Key Laboratory for Special Pathogens Prevention and Control, Hunan Province Cooperative Innovation Center for Molecular Target New Drug Study, University of South China, Hengyang, China

**Keywords:** mycoplasma, immune evasion, antigenic variation, oxidative stress, neutrophil extracellular traps

## Abstract

Mycoplasmas are a large group of prokaryotes which is believed to be originated from Gram-positive bacteria via degenerative evolution, and mainly capable of causing a wide range of human and animal infections. Although innate immunity and adaptive immunity play crucial roles in preventing mycoplasma infection, immune response that develops after infection fails to completely eliminate this bacterium under certain circumstances. Thus, it is reasonable to speculate that mycoplasmas employ some mechanisms to deal with coercion of host defense system. In this review, we will highlight and provide a comprehensive overview of immune evasion strategies that have emerged in mycoplasma infection, which can be divided into four aspects: (i) Molecular mimicry and antigenic variation on the surface of the bacteria to evade the immune surveillance; (ii) Overcoming the immune effector molecules assaults: Induction of detoxified enzymes to degradation of reactive oxygen species; Expression of nucleases to degrade the neutrophil extracellular traps to avoid killing by Neutrophil; Capture and cleavage of immunoglobulins to evade humoral immune response; (iii) Persistent survival: Invading into the host cell to escape the immune damage; Formation of a biofilm to establish a persistent infection; (iv) Modulation of the immune system to down-regulate the intensity of immune response. All of these features increase the probability of mycoplasma survival in the host and lead to a persistent, chronic infections. A profound understanding on the mycoplasma to subvert the immune system will help us to better understand why mycoplasma is so difficult to eradicate and ultimately provide new insights on the development of therapeutic regimens against this bacterium in future.

## Introduction

Mycoplasmas (class *Mollicutes*) are the smallest and simplest self-replicating organisms. Due to the lack of a rigid cell wall, this bacterium is only bound by an outer structure including capsule, adhesive structure and adhesion-related proteins, as well as a unit membrane. Although the basic structure is simpler than the common Gram-negative bacteria, there is a complex cross-talk between mycoplasma and the host immune system involving mycoplasma-induced non-specific and specific immune responses. Similar to other microorganisms, at the beginning of infection, the innate immune response, consisting mainly of innate immune molecules and innate immune cells, is non-specific but plays a critical role in the defense against this microbe. Innate Immune cells, such as neutrophils, macrophages, and natural killer cells, not only have the capacity to recognize pathogen-related molecular patterns (PAMPs) of mycoplasma via toll-like receptors (Shimizu et al., 2005), but also can kill these microorganisms. First of all, phagocytizing neutrophils and monocytes/macrophages inevitably yields oxidative bursts that are elicited by bacterial infections, leading to the release of reactive oxygen species (ROS). ROS are essential participants of various innate immune cell responses against microorganisms, including oxidative radicals such as superoxide, hydroxyl radicals, H_2_O_2_, and organic hydro-peroxides (Nguyen et al., 2017). The responsive production of these molecules is highly toxic and brings about severe, even irreversible, damage to bio-macromolecules, such as DNA, proteins, and lipids of invading pathogens (Storz and Imlay, 1999). Additionally, neutrophils act as the first line of innate immune defense against pathogenic microbes. In addition to their phagocytic activity, the release of neutrophil extracellular traps (NETs) has been identified as an alternative mechanism of killing invading microbes (Brinkmann et al., 2004; Papayannopoulos and Zychlinsky, 2009). Besides, the production of a variety of cytokines and the complement system also plays non-negligible roles in innate and adaptive immune reactions. Cytokines are key reactive and modulatory molecules participating in innate and adaptive immune response, such as the recruitment and activation of immune cells and the induction of initial cell differentiation. The complement system not only constitutes part of the innate immune system but also is one of the important tactics by which antibodies exert their immune effects. In the late stage of infection, the adaptive immune response, whose specific participant cells and molecules are different types of T/B lymphocytes and antibodies, promoting further elimination of the invading pathogen.

**FIGURE 1:**
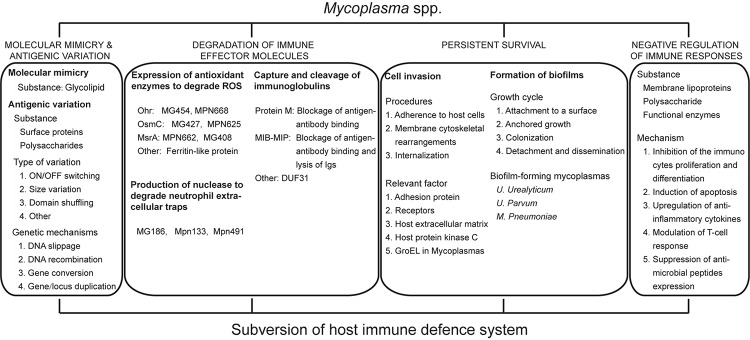
Molecular mechanism of human pathogenic mycoplasmas to evade the host Immune attack.

In the face of powerful immune system, despite limited biosynthetic capabilities, mycoplasmas still have the ability to propagate and survive within the host for a long period of time after invading an appropriate host. To maintain their survival and persistent infection, it is very likely that mycoplasma have evolved rather sophisticated mechanisms to resist to coercion by the host immune system. In addition to molecular mimicry and antigenic variation, which was first described and is widely accepted, we will also present other novel strategies that mycoplasma have evolved, including defense against oxidative stress, degradation of NETs, capture and cleavage of immunoglobulins, cell invasion, the formation of biofilms and negative regulation of the immune response (Figure 1), which will provide new insights in to cross-talk between mycoplasma and the host.

## Molecular Mimicry and Antigenic Variation

It is universally accepted that molecular mimicry and antigenic variation are proficient mechanisms that several bacteria have evolved to cope with the host defense system. Molecular mimicry refers to the same antigenic epitope exists between different mycoplasmas and the host cells, closely associated with autoimmune diseases. The most frequently discussed examples related to molecular mimicry is the Guillain–Barré syndrome, which is induced by *M. pneumoniae* infection with a lipid structure that results in the enhancement of galactocerebroside-specific antibodies level from patients. This cross-reactivity is relevant for autoimmunity and might be exploited by mycoplasmas to avoid recognition by the adaptive immune system (Kusunoki et al., 2001). Although molecular mimicry is largely accepted as the way to escape the immune surveillance, it is rarely occurred in other mycoplasma species, therefore its role in mycoplasma infection needs further investigation.

Antigenic variation, also known as phenotypic switching, originates from genetic mutations (occurring at a frequency of 10^–2^–10^–5^) that occur at a higher rate than what is considered the normal mutation rate (10^–6^–10^–8^). Although mycoplasma species lack a rigid cell wall, lipoproteins function as the major constituents that come into contact with the host surroundings (Christodoulides et al., 2018). Many of these proteins have been demonstrated to undergo antigenic variation, such as on/off switching, size variation, and domain shuffling, among others, to change the antigenic components on their cell surface to generate “hetero-types” that allow for bacteria to avoid recognition and clearance by host immune cells that predominantly eliminate “homo-types.” A large number of human and animal mycoplasma species have been demonstrated to have the capacity to undergo antigenic variation so that these microbes can elude recognition by the host humoral immune system.

As early as 2010, a classic review reported that most mycoplasma species could generate antigenic variation, including *Mycoplasma genitalium, M. penetrans, M. hominis*, *M. hyorhinis*, *M. gallisepticum, M. capricolum* subsp. *capricolum*, *M. pulmonis, M. bovis, M. agalactiae, M. synoviae, Ureaplasma parvum*, and *U. urealyticum* (Citti et al., 2010). In that review, the authors elaborated the genetic mechanisms, which included DNA slippage, DNA recombination, gene conversion and occasionally gene or locus duplication. The authors found that one or more of these mechanisms could concomitantly result in diverse antigenic variation and be implicated in many relevant gene families, including the *mpl*, *mgp*, *lmp*, *mba*, *vip*, *vihA*, *vmc*, *vsa*, *vsp*, and *vpma* gene families. However, these gene families have never been reported to exist in some of the important pathogenic mycoplasma species, such as *M. pneumoniae* and *M. fermentans*. Therefore, here we will further supplement these new insights into human pathogenic mycoplasma concerning antigenic variation.

One study demonstrated that proteins encoded by MPN536 in *M. pneumoniae* strain FH and MG359 in *M*. *genitalium* that are homologous to RuvB (Holliday junction migration motor protein), which contributes to homologous recombination, a key genetic event in the production of antigenic variation, possessed divalent cation- (Mg^2+^ or Mn^2+^) and ATP-dependent DNA helicase activity (Estevão et al., 2011). Compared with RuvB_*FH*_, RuvB_*M*__129_ displayed a faint DNA unwinding activity with a single amino acid residue change, which was implicated in the difference in DNA helicase activity. These results indicate that *M. pneumoniae* and *M*. *genitalium* could undergo antigenic variation via homologous recombination. For *M*. *genitalium, mgpB* and *mgpC* encoding P140/MG191 and P110/MG192, respectively, were indispensable for the adherence and motility of this organism; Wood and colleagues successfully established a Macaca nemestrina model of *M*. *genitalium* infection and observed that sequence variation in region B of *mgpB* occurred after 8 weeks (Wood et al., 2013). Rabbit antibodies that reacted to the MgpB region B variant peptide sequence were diminished. Moreover, MG192 has been recently demonstrated to be a major adhesion recognized by human sialic acid receptors (Aparicio et al., 2018). Furthermore, structural analysis also demonstrated that the antigenic region of the MG192 protein underwent programed variation. Hence, it is usually considered that high-frequency antigenic variation occurs only in the variable regions of MG191/MG192. Interestingly, *mgpB*/*C* phase variants lacking adherence properties were still able to evade killing by antibodies and complement through avoiding antibody recognition of their variable regions (Burgos et al., 2018). This result indicates that *M. genitalium* could also escape antibody-mediated killing by virtue of altering its conserved C-terminal domain. Before this, one study demonstrated that the mutation frequencies of *mgpB*/*C* were closely associated with overexpression of RecA, which also has been shown to exhibit a slight DNA repair activity (Burgos et al., 2012). In addition, RecA has been shown to be essential for σ^20^-mediated horizontal gene transfer in *M. genitalium* (Torres-Puig et al., 2018). These data hint that some mycoplasma species may be able to generate diverse antigenic peptides through high-frequency, random mutations in maintenance and repair systems to avoid challenges from host defense mechanisms. Later, this group also confirmed that the MG428 protein was an indispensable factor that positively regulated the expression of some genes, including *recA*, *ruvA*, *ruvB* and ORF2, and induced homologous recombination, generating antigenic and phase variation (Burgos and Totten, 2014). Again, these findings attract great attention from a large number of scholars. Ma et al. have isolated *M. genitalium* strains from two male NGU (non-gonococcal urethritis) patients and, through sequence comparisons, observed a striking phenomenon that extensive variation and rapid shifts occurred in the V4 and V6 regions of the MG192 sequence (Ma et al., 2014). Further exploration of MG192 by infecting two chimpanzees with a single cloned *M. genitalium* type strain, G37, supported that MG192 variation is a principal strategy that this organism has adopted to evade host immune defenses (Ma et al., 2015).

In addition to surface proteins, some polysaccharides also appear to generate antigenic variation. Many, but not all, gram-positive and gram-negative bacterial species, such *as Neisseria gonorrhoeae*, *Helicobacter pylori*, and *Campylobacter jejuni*, are capable of yielding polysaccharides with phase variations that involve on/off switching (Banerjee et al., 2002; Sanabria-Valentín et al., 2007; Parker et al., 2008). More recently, increasing evidence has confirmed that some wall-free mycoplasma species, such as *M. penetrans, M. pulmonis, M. pneumoniae, M. mycoides* subsp. *mycoides, M. mycoides* subsp. *capri* serovar *capri, M. mycoides* subsp. *capri* serovar LC*, M. leachii, M. capricolum* subsp. *capripneumoniae, M. capricolum* subsp. *capricolum* and *M. agalactiae*, also possessed the ability to synthesize and secrete capsular and/or exopolysaccharides attached to their membrane surfaces (Neyrolles et al., 1998; Daubenspeck et al., 2009; Bertin et al., 2013, 2015; Simmons et al., 2013; Gaurivaud et al., 2016). In one experiment, the secretion of cell-attached β-(1→6)-glucan by the ruminant pathogen *M. agalactiae* was found to be off-switching when the coding sequence of a polyG tract synthase gene, *gsmA*, underwent size variation (Gaurivaud et al., 2016). Compared to wild-type strains, the glucan phase-variation mutant showed a low susceptibility to killing by serum. Whether similar mechanisms exist in human mycoplasmas remains to be further discussed. These findings open another effective avenue to the better understanding antigenic variation and emphasize that the mechanism employed by mycoplasma is of great significance to the evasion of host immune defenses.

## Overcoming the Immune Effector Molecules Assaults

### Defenses Against Oxidative Stress

In addition to intracellular oxidative damage, host immune cells, such as PMNs and monocytes/macrophages, release substantial ROS including superoxide anions, hydrogen peroxide and hydroxyl radicals after mycoplasma activates surface receptors. The locally high concentration of ROS contributes to the elimination of invading pathogens. To overcome oxidative stress caused by ROS, which serve as part of the host’s innate immune response, it is speculated that mycoplasma may have evolved a protective strategy to detoxify oxidizing agents, such as superoxide dismutase (SOD), catalase (Cat) and alkyl hydroperoxide reductase (AhpR). However, it was previously shown that *M. pneumoniae* and *M. penetrans* lack the genes that encode these enzymes. Oddly, high thioredoxin reductase system activities were found in some human pathogenic mycoplasma species, including *M. pneumoniae*, *M*. *fermentans* and *M. penetrans* (Ben-Menachem et al., 1997), suggesting that these organisms may be resistant to the effects of endogenous, or even exogenous, oxidative molecules.

In other pathogenic bacteria, the organic hydroperoxide resistance (Ohr) protein and osmotically inducible protein C (OsmC) have been found and demonstrated to be specifically associated with the detoxification of organic peroxides to the corresponding alcohols (Mongkolsuk et al., 1998; Lesniak et al., 2003; Meunier-Jamin et al., 2004). By sequence comparison, the genes for Ohr and OsmC have also been identified in some Mollicute genomes, suggesting that mycoplasma possess a novel mechanism of protection from oxidative assault. The OsmC superfamily is composed of OsmC, Ohr and a group of structurally related proteins with unknown functions (subfamily III). In Mollicutes, the Ohr protein was first found in *M. gallisepticum* (Jenkins et al., 2008), and the phylogenetic tree clearly showed that the *mga1142* gene belonged to the Ohr subfamily and was homologous to other members of the *M. pneumoniae* phylogenetic group (containing only *M. pneumoniae* and *M. genitalium*). However, no homologs of the Ohr proteins were found in any other mycoplasma species, indicating that this protein may be unique to the *M. pneumoniae* phylogenetic group. Further analysis has shown that the products of *M. genitalium mg427* (Zhang and Baseman, 2014), *M. pneumoniae mpn625* (Jenkins et al., 2008), and *M. gallisepticum mga0252* (Jenkins et al., 2008) exhibit more similarity to OsmC, while the products of *mg454* (Saikolappan et al., 2009), *mpn668* (Chen et al., 2018), and *mga1142* (Jenkins et al., 2008) show more similarity to Ohr. This observation is consistent with experimental results; However, the relative size and shape of the *mpn625* active site (a protein from *M. pneumoniae* that is homologous to *mga0252* and belongs to the OsmC subgroup III) is most comparable to that of *mga1142*. To date, the functions of *mpn625* and *mga252* have not yet been determined. The protein encoded by *Mpn625* was predicted to possess peroxidase activity and overall structural similarity to other members of the OsmC superfamily because of the position of its cysteine residues. Subcellular location of Ohr/OsmC demonstrated that these proteins were prominent in both the cytoplasmic and/or membrane fractions. By computer modeling, we can infer that Ohr/OsmC is a homodimer containing two highly catalytic redox-reactive cysteines located at the monomer interface on opposite sides of the molecule, like a hydrophobic pocket, designed to swallow and neutralize non-organic or organic hydro-peroxides, in particular, *tert*-butyl hydroperoxide (tBOOH) based on the FOX assay, and create a disulfide bridge (Jenkins et al., 2008). However, the mechanisms of OsmC/Ohr regulation by mycoplasma under oxidative stress are not completely understood. It seems that the Ohr protein is generally upregulated in the presence of organic peroxides, while OsmC is found to be upregulated under osmotic shock and ethanol stress conditions. Nevertheless, this is not the case because expression of both *mga1142* and *mg454* were unchanged under oxidative stress conditions, but that of *mpn668* (Jenkins et al., 2008; Saikolappan et al., 2009; Chen et al., 2018). One report showed that the product of *mg427* (OmsC) was significantly down-regulated by osmotic shock (Zhang and Baseman, 2014). More intriguingly, contrary to *mg427*, the expression of *mg454* was up-regulated by heat shock, whereas the upstream promoter region of this gene lacks the obvious CIRCE (controlling inverted repeats of chaperone expression) element to which HRCA (heat shock regulation at CIRCE) binds for the repression of gene expression for transcriptional heat shock response (Saikolappan et al., 2009). These data suggest that mycoplasma possess a novel pattern to regulate the expression of these two proteins. Furthermore, a putative -10 site and −35 site in the promoter region of σ^70^ and σ^*E*^ were identified in *mg427* and *mga1142*, respectively, yet other data indicated that the expression of *mpn668* may regulate the OhrR homolog encoded by *mpn314* in a manner that is distinctly different from other mycoplasma Ohr proteins. Although these data show that the Ohr/OsmC proteins can resist damage by ROS, whether they play a primary role in escaping killing by oxidative stress remains unclear.

As increasing numbers of antioxidant proteins are confirmed, the role of Ohr/OsmC becomes increasingly less important. Furthermore, similar findings demonstrated that *M. pneumoniae* and *M. genitalium* encode a homologous protein known as peptide methionine sulfoxide reductase (MsrA), which is an antioxidant repair enzyme that catalyzes the reduction of methionine sulfoxide [Met(O)] residues in proteins to methionine (Dhandayuthapani et al., 2001). In addition, in 2015, our group found that a ferritin-like protein with ferroxidase activity in *U. urealyticum* could impair harmful oxidative production *in vitro* (Dai et al., 2015). On the basis of these known data, the mechanism employed by mycoplasma in reaction to oxidative stress plays an important role in mycoplasma survival within host. Even though mycoplasma can degrade ROS, this ability does not mean that the host will succumb to the organism because ROS can also act as a messenger molecule to activate other immune responses, such as NETs, which are a hotspot for recent research.

### Degradation of Neutrophil Extracellular Traps

Neutrophils play a vital role in antimicrobial defense, constituting the first line of the innate immune system. In addition to their phagocytic activity, NETs have been recently described as an alternative mechanism of scavenging invading bacteria (Papayannopoulos and Zychlinsky, 2009). NETs are web-like structures that consist of chromatin undergoing histone citrullination, chromatin decondensation and spreading, and a few antimicrobial granule proteins, such as elastase and myeloperoxidase, which are released after cell membrane disruption. Under conditions of high local concentrations, NETs can efficiently entrap and kill invading pathogens including *Escherichia coli*, *Salmonella typhimurium*, and *Shigella flexneri*, among others (Brinkmann et al., 2004; Grinberg et al., 2008). On the other hand, some pathogens are known to have evolved a variety of strategies to protect them from killing by NETs, such as cell surface structure changes, interference in NETs formation, and production of nucleases that degrade DNA components of NETs (Storisteanu et al., 2017).

It is well recognized that neutrophils play a limited role against mycoplasma infection, and co-incubation of mycoplasma with neutrophils has no evident impact on the growth of the mycoplasma (Howard and Taylor, 1983). Some studies demonstrated that various mycoplasma species, including human mycoplasma species, possessed multiple membrane-associated nucleases to degrade NETs (Razin et al., 1964; Minion et al., 1993; Paddenberg et al., 1998). Therefore, these findings synergistically and strongly imply that, similar to other bacteria, mycoplasma may also possess mechanisms to secrete effective nucleases to evade killing by NETs. However, no specific mechanism has been elaborated. Subsequently, two study groups identified and cloned a nuclease gene (*mnuA*) from *M. pulmonis* with significant homology at the amino acid level to an uncharacterized protein (P01_orf474) of *M. pneumoniae* (Jarvill-Taylor et al., 1999). The gene was also homologous to a gene in *M. pneumoniae*, *M. penetrans*, and *U. urealyticum*, but not *M. genitalium.* MnuA, encoded by *mnuA*, is a 51-kDa protein with a single cysteine residue that was considered to be a non-specific nuclease anchored in the cell membrane. Subsequently, *M. hyopneumoniae mhp379* (Schmidt et al., 2007), encoding a putative lipoprotein, was demonstrated to be MnuA and using the GenBank database, orthologous sequences were found to be present in *M. genitalium* and *M. synoviae* in addition to those mycoplasma mentioned above. Moreover, a number of studies discovered that most mycoplasma homologs of *mhp379* were consistently located upstream or downstream of an ABC transporter system equipped with a hydrophobic amino-terminal signal sequence along with a prokaryotic lipoprotein cleavage site. Additionally, it was uncovered that the SNc regions of mhp379 and its homologs were similar to the thermostable nuclease secreted by *S. aureus* (Schmidt et al., 2007), the majority of which take along with three conserved active catalytic site residues (arginine, glutamate, and arginine) and Ca^2+^ binding site residues (aspartate, aspartate, tyrosine). Following mhp379, Li et al. identified a membrane-associated lipoprotein, MG186 that displayed sugar-non-specific endonuclease and exonuclease activity dependent on Ca^2+^ (Li et al., 2010). In general, most mycoplasma nucleases possess endonuclease and/or exonuclease activity that is strictly reliant on the presence of Ca^2+^ and/or Mg^2+^, existing in a membrane-bound or secreted form. However, differences also exist between nucleases from distinct mycoplasma species, as detailed in Table 1, which may result from the regressive process of mycoplasma species.

**TABLE 1:** Nuclease characteristics in human pathogenic mycoplasmas.

**Species**	**Gene or protein**	**Divalent cation**	**Inhibitory agent**	**Subcellular location**	**Nuclease activity**	**References**
*M. penetrans*	P40	Ca^2+^, Mg^2+^	EDTA, EGTA, Zn^2+^, and so on	Secreted, membrane-associated	Endo-	Bendjennat et al., 1997, 1999
*M. genitalium*	MG186	Ca^2+^	Zn^2+^, Mn^2+^, EDTA, EGTA	Surface-exposed	Endo-/exo-	Li et al., 2010
*M. pneumoniae*	Mpn491	Mg^2+^	Zn^2+^	Secreted	Endo-/exo-/phosphatase	Yamamoto et al., 2017

Despite the fact that mycoplasma nucleases are known to utilize host cell nucleic acid precursors to contribute to their growth and survival, it is yet unclear what role, if any, mycoplasma nucleases play in the protection against host immune defensive NETs. As a result, interactions between mycoplasma and neutrophils are a recurring theme that has attracted many scientists. Approximately a decade ago, *M. pneumoniae* was demonstrated to produce a membrane-associated nuclease, Mpn133 (Somarajan et al., 2010), with a glutamic acid-lysine-serine-rich region, but no enzymatic activity. Recently, by means of transposon insertion, a protein identified from *M. pneumoniae*, Mpn491, was shown to exhibit endonuclease and exonuclease activity. Mpn491 was found to be mainly responsible for the evasion of antimicrobial activities of NETs *in vivo* and *in vitro*, and mutation of this protein had little effect on the growth of this organism (Yamamoto et al., 2017). Meanwhile, Mpn491 was reported to possess an amino acid sequence similar to the glutamic acid-lysine-serine region. Taken together, these results clearly suggest that relevant nuclease production is an indispensable mechanism for mycoplasma to circumvent NETs assault and provide a perspective for the reason that some mycoplasmas localize perinuclear regions after invading into the host cells.

### Capture and Cleavage of Immunoglobulins

As an important effector mediating fluid immunity, antibodies belong to immunoglobulins (Igs), consisting of Fab and Fc regions, and can specifically recognize and bind to corresponding antigens via the Fab region and elicit a series of biochemical reactions, such as neutralization of antigens, activation of complement, and combination with Fc receptors, among others. On the other hand, many important pathogenic bacteria, such as *S. aureus* and streptococcal species, have evolved several sophisticated means to elude or disrupt Ig-mediated immune defense, such as binding to Fv region of Igs to evade immune clearance (Woof, 2016). Additionally, increasing research has reported that similar strategies are involved in mycoplasma infection. Initially, scores of scientists discovered that *Ureaplasma* spp. was able to cleave human IgA1, but not other IgA sub-classes, at the hinge region between its Fab and Fc fragments (Robertson et al., 1984; Kapatais-Zoumbos et al., 1985). Therefore, *Ureaplasma* spp. is predicted to have serine protease activity; however, no genes encoding the protease have been identified to date. In addition, a survey validated that protease activities were able to be found in any of human or animal mycoplasma species (Watanabe et al., 1985). In the human urethral pathogen *M. genitalium*, an Ig binding protein referred to as protein M has a strong affinity for human and non-human IgG, predominantly binding to conserved parts of the VL domain of the light chain, which blocks antigen-antibody binding (Grover et al., 2014). Moreover, in comparison to other mycoplasma strains, there are homologs of protein M present in *M. pneumoniae*, *M. gallisepticum*, and *M. iowae.*

More recently, a two-protein system comprised of mycoplasma Ig binding protein (MIB) and mycoplasma Ig protease (MIP), referred to as the MIB-MIP system, was described in *M. mycoides* subsp. *capri* (Arfi et al., 2016). MIB is the protein that may be capable of binding closely to the Fv region of all types of IgG to form the MIB-IgG complex, which recruits MIP and enables the serine protease activity. Unlike other known proteases targeting at the hinge region, the MIP cleavage site is located between the VH and CH1 domains. Interestingly, this study revealed that the MIB-MIP system also exists in the majority of animal and human mycoplasma species, including *U. urealyticum, U. parvum*, and *M. hominis*, but not in some mycoplasma that possess protein M that is structurally bound to MIB, with the exception of *M. gallisepticum.* Furthermore, those investigators have detected a gene annotated as *duf31* whose products contained a Pfam domain that was initially thought to be MIP and MIP paralogs but was found not to be homologous with MIP and remained elusive, though its conserved amino acid sequence indicated that it may possess serine protease activity (Arfi et al., 2016). The *duf31* gene appears to be widespread in the animal and human pathogenic mycoplasma species, while absent from plant pathogenic species. It is worth noting that there are some mycoplasma, such as *M. pneumoniae* and *M. genitalium*, which are lacking the MIB-MIP system, but possess both protein M and DUF31 encoding genes. Thus, it is surmised that protein M functions in pair with the predicted DUF31 domain and, hence, may play a similar role in circumventing host Ig-mediated defense against the MIB-MIP system. Finally, the Ig binding protein-Ig protease system is a new avenue that protects mycoplasma against the host immune response and is contributing to a better understanding of mycoplasma immune evasion.

## Persistent Survival

### Cell Invasion

It was generally recognized that mycoplasma were strict prokaryotes that remained extracellular or adhered to the surface of epithelial cells without the ability to invade into host cells. With the rapid development of modern biotechnology, for example confocal laser scanning microscopy has been instrumental in distinguishing invasion from adherence in most pathogens, which was often confused in the past. A large number of invasive bacterial pathogens appear to enable their invasion or internalization into non-phagocytic cells.

*Mycoplasma penetrans*, originally isolated from the urogenital tracts of AIDS patients, was the first identified to have the capacity to penetrate into mammalian cells and as such, was named based on this ability. With the increasing depth of research, scientists revealed that a few other intact mycoplasma that colonize the respiratory and/or urogenital tract, such as *M. fermentans*, *M. hominis*, and *M. pneumoniae*, can facultatively enter into host cells and localize throughout the cytoplasmic and perinuclear regions (Taylor-Robinson et al., 1991; Baseman et al., 1995; Rosengarten et al., 2000). Using gentamicin resistance assays, dozens of studies validated that intercellular mycoplasma could avoid killing by antibiotics. Several other investigators demonstrated that certain mycoplasmas also possessed similar invasive characteristics that pointed toward non-phagocytic and/or even phagocytic cells in both *in vivo* and/or *in vitro* models (Table 2; Dusanić et al., 2009; Groebel et al., 2009; Kornspan et al., 2010; van der Merwe et al., 2010; Hegde et al., 2014, 2015; Zhang et al., 2015). These data implies that *Mycoplasma* spp. in general may possess the capability of invading host cells and hence have acquired a unique way of resisting host immune defenses and specialized antibiotic therapies, and achieving nearly unlimited nutrients for establishing chronic, persistent infections.

**TABLE 2:** Intracellular localization of human pathogenic *Mycoplasmas*.

**Species**	**Phylogenetic group**	**Disease/pathology**	**GroEL presence**	**Target cells**	**References**
*M. penetrans*	Pneumoniae	AIDS-associated	Yes	HeLa	Tarshis et al., 2004
				Molt-3	Salman et al., 1998
*M. genitalium*	Pneumoniae	Genital infections	Yes	Vero	Jensen et al., 1994
				Vaginal and cervical epithelial cells	Blaylock et al., 2004
				HeLa and EM42	Ueno et al., 2008
*M. pneumoniae*	Pneumoniae	Respiratory disease	Yes	A549	Yavlovich et al., 2004b
*M. fermentans*	Hominis	Genital infections	Yes	HeLa	Yavlovich and Rottem, 2007

Consistent with similar observations in other microorganisms, adherence has a drastic impact on cell invasion. However, the precise mechanism is poorly understood. During the interaction between mycoplasma and host cells, it seems that some cell constituents of mycoplasma and the host extracellular components are indispensable. Yavlovich et al. (2001, 2004a) and Yavlovich and Rottem (2007) have validated that *M. fermentans* can bind to host extracellular matrix proteins, such as plasminogen, fibronectin, heparin, laminin and collagen. Among interactions, the binding of plasminogen to *M. fermentans* is intensive, and it has been clearly shown that the invasiveness of *M. fermentans* is attributed to the activation of bound-plasminogen to plasmin, suggesting that it is possible that the proteolytic modification of these organisms and/or host cell surface proteins enables internalization. Additionally, this mycoplasma-binding plasminogen phenomenon has also been observed in *M. pneumoniae* and *M. gallisepticum* (Yavlovich et al., 2004b; Fürnkranz et al., 2013). Indeed, some studies have demonstrated that both the binding and the invasive capacity of intact mycoplasma cells treated with trypsin or proteinase K were significantly reduced. However, contrary results have also been reported in other studies (van der Merwe et al., 2010). This difference may be the result of different protease concentrations and different reaction conditions. Intriguingly, another study recently discovered that α-enolases located on the mycoplasma cell surfaces mediated plasminogen binding based on the formation of hydrogen bonds, indicative of its role in facilitating host cell invasion (Chumchua et al., 2008; Song et al., 2012).

Cell invasion is a multifactorial process including the involvement of receptors, functional genes, kinases and cytoskeletal rearrangements that are mediated by microtubules and/or microfilaments (Borovsky et al., 1998). In this sense, it is reasonable to suspect that receptors on the surface of host cells are indispensable. But, few receptors involved in this process have been identified. Until recently, Shimizu et al. (2014) reported that TLR4 was the key receptor in macrophages involved in the endocytosis of *M. pneumoniae*, indicating that TLRs may play a critical role in mycoplasma cell invasion. Another interesting finding is that the invasive ability of low-passage mycoplasma strains is obviously higher than that of high-passage strains (Winner et al., 2000; Vogl et al., 2008), suggesting that the loss of invasive ability observed in some mycoplasma species may be due to the accumulation of gene mutations/deletions during passage. It also has been demonstrated that the internalization process of *M. penetrans* is implicated in the activation of protein kinase C, the induction of tyrosine phosphorylation of a 145-kDa host cell protein and the presence of a 42-kDa mycoplasma membrane lipoprotein (Rottem and Naot, 1998). In addition, the high invasiveness property of *M. penetrans* appears to be closely associated with GroEL, a heat shock protein and chaperone stemming from lateral gene transfer (Clark and Tillier, 2010). There are some experimental evidences indicating GroEL present in *M. fermentans* (Søndergård-Andersen et al., 1990) and *M. suis* (Hoelzle et al., 2007) and GroEL has demonstrated to be maintained in the genomes of four important etiological mycoplasma species other mycoplasmas species, including *M. penetrans*, *M. pneumoniae*, *M. genitalium*, and *M. gallisepticum* (Clark and Tillier, 2010), suggesting the role of GroEL in invasion process of mycoplasma. Additionally, the dependence of mycoplasma internalization on durable infection and temperature has been described in some cases, which may be a result of the fact that durable infection and appropriate temperature increase the affinity of mycoplasma to host cells. Overall, although the specific mechanisms by which mycoplasma invade host cells are only poorly understood and still need to be elucidated, it is undeniable that *Mycoplasma* spp. can escape the host immune system and survive within host cells. Furthermore, it is likely that cell invasion enables the bacteria to pass through cell barriers (e.g., the mucosal epithelium) into tissues or organs and contributes to diverse diseases.

### Formation of Biofilms

Biofilms can be defined as a functionally heterogeneous congregation of micro-colonies or single cells encapsulated with self-produced polymeric matrixes composed of polysaccharides, lipids, proteins and extracellular DNA (eDNA) originating from cell autolysis. Compared with planktonic cells, these matrixes form what is widely regarded as a protective mechanism for the majority of pathogenic and non-pathogenic bacteria or fungi that produce them, allowing these organisms to be more resistant to surrounding stresses, such as antibiotics, antibodies and phagocytes/non-phagocytes (Roilides et al., 2015; Kumar et al., 2017). The general procedure of biofilms development has been described to embody a number of mutual characteristics, primarily including (i) attachment to a surface, (ii) anchored growth, (iii) colonization, (iv) detachment and dissemination. Moreover, with only a limited genome, whether mycoplasma could develop such complex and powerful biofilms remains to be elaborated. It is intriguing to speculate that mycoplasma may form biofilms by virtue of a simple and a stochastic mechanism.

Previously, McAuliffe et al. (2006) examined the ability of different mycoplasma species to form biofilms and found that some animal mycoplasma species shared the ability to form a notable biofilm attached to inert surfaces and thus were more resistant to antimicrobials as well as physical stresses such as heat and desiccation. In human mycoplasma species, *M. pneumoniae* can also yield a characteristic volcano-like biofilms on glass or polystyrene surfaces (Kornspan et al., 2011). In that experiment, adherence and/or biofilms formation were sharply inhibited by the anti-P1 polyclonal monospecific antibodies, neuraminidase or sialyllactose, which could be explained by the fact that P1 can bind to human sialic acid as its receptors (Aparicio et al., 2018). These data indicate that cytoadherence, this step is an essential prelude to biofilms formation. Furthermore, compared with *M. pneumoniae* strain UAB PO1, the M129 strain had a weak ability to form biofilms (Simmons et al., 2013). It is obvious that there are a few inter- and intra-species differences existing and remaining to be elucidated. Surprisingly, a pilot study observed that pre-treatment with catalase confers an advantage on accelerating the development of *M. pneumoniae* biofilms (Simmons and Dybvig, 2015), but the reason is unknown.

In addition to the *M. pneumoniae*, two of four *U. urealyticum* human isolates and both *U. parvum* human isolates were shown to be able to develop biofilms that were less affected by most macrolides, with the exception of clarithromycin, compared to planktonic cells (García-Castillo et al., 2008). Compared to clarithromycin, azithromycin, another antibiotic with better permeability, has been found to have a specific capacity to avert biofilms formation by inhibiting the production of alginate and blocking quorum sensing signaling of *P. aeruginosa* (Nalca et al., 2006). However, azithromycin has not been applied in mycoplasma studies. By gene comparison, no genes modulating biofilms formation that are homologous with those of other well-known bacteria, such as *esp* of *Enterococcus faecalis* or *bap* of *S. aureus*, have been found in *Ureaplasma* spp. In summary, biofilms formation is a vital mechanism to escape host immune responses and to resist ambient threats, making antibiotic treatment formidable.

## Negative Regulation of Immune Responses

Apart from the host generating a wide range of anti-mycoplasma immune responses, *Mycoplasma* spp. also exert a series of non-specific immunosuppressive effects upon the host immune cells. As early as 1989, evidence provided by Foresman and colleagues showed that mycoplasma or their products could induce immune disability by inhibiting the activation of T/B lymphocytes (Simberkoff et al., 1969; Foresman et al., 1989). *M. arginini* was the first reported to be capable of inhibiting the growth of lectin-stimulated T cells as a result of a lymphocyte blastogenesis inhibitory factor (LBIF), which was purified and identified as arginine deiminase (Sugimura et al., 1990). In addition to *M. arginini*, arginine deiminase was also characterized in *M. hominis* and *M. oral* but not *M. pneumoniae* or *M. fermentans*, and it has been shown to prevent lymphoid cell division and proliferation (Simberkoff et al., 1969; Foresman et al., 1989), suggesting that it may be one of the mechanisms by which mycoplasma negatively regulates the immune system (Gill and Pan, 1970). Although similar arginine deiminase was also found in *M. penetrans* (Gallego et al., 2012), but its function on host immune system needed further investigation.

Fusing with lymphocytes and induction of apoptosis in immunocyte is another step in negative-regulation of the immune system. For example, the *M. fermentans* incognitus strain isolated from an AIDS patient could fuse to CD4^+^ T cell lines and human peripheral blood lymphocytes, yielding cytocidal effects on these immune cells; However, the fusion process was not associated with microtubules or actin filaments, which distinguishes this process from the process of cell invasion by mycoplasma (Franzoso et al., 1992; Dimitrov et al., 1993). Until a decade ago, Into et al. (2002) had highlighted that mycoplasma-derived lipoproteins were able to induce innate immune cell (monocytes/macrophages) and lymphocyte death for the first time. With further exploration, several later studies found that lipoproteins, also known as lipid-associated membrane proteins (LAMPs), such as P48 of *M. fermentans*, were capable of inducing immunocyte apoptosis (Hall et al., 2000; Liu et al., 2019).

In addition to their roles as inhibitor of the function of lymphocytes and monocytes/macrophages, mycoplasmas or their components also play an important role in promoting the secretion of anti-inflammatory cytokines, and one of the typical examples is IL-10. *M. hominis* was recently shown to induce dendritic cells (DCs) to secrete IL-10, but could not effectively activate NLRP3 inflammasome (Goret et al., 2017). And evidences provided by our group also confirmed that *M. pneumoniae* extracts could induce DCs secretion of IL-10, and the active component was capsular polysaccharides (Liu et al., 2013). Not only that, Noda-Nicolau et al. (2016) discovered that two important genital mycoplasma, *U. urealyticum* and *M. hominis*, shared the capacity to upregulate the anti-inflammatory cytokines IL-10 and IL-13, but they rarely affected the levels of pro-inflammatory cytokines. Recently, community acquired respiratory distress syndrome (CARDS) toxin, a unique bacterial ADP-ribosylating and vacuolating toxin produced by *M. pneumoniae*, has been found to induce cytokine suppression through continuous decrease of IL-17 and IFN-γ, increase of IL-4/IFN-γ ratio and then polarization of the type-2 phenotype T-cell response, leading to a partial anergy of the immune responses mediated by T cells (Maselli et al., 2018). Thus, the initial release of anti-inflammatory cytokines and modulation of the T-cell response cell may indirectly contribute to immune evasion.

In recent years, hydrogen sulfide (H_2_S), a deleterious endogenous gasotransmitter, has been shown to attenuate the inflammatory response under certain conditions (Benedetti et al., 2017). Großhennig et al. (2016) characterized a cysteine desulfurase, HapE from *M. pneumoniae*, and demonstrated that it was a bifunctional enzyme that could produce H_2_S, which was considered a virulent factor that can induce the lysis of erythrocytes. Although it is not clear whether HapE directly affects the host immune system, H_2_S has been shown to exhibit anti-inflammatory features and could inhibit the production of MCP-1, which is a key pro-inflammatory factor in the recruitment of monocytes to an infection site (Benedetti et al., 2014). On the other hand, Francesca et al. inferred that H_2_S exerted anti-inflammatory effects by inhibiting the activation of the TLR-mediated nuclear factor-κB (NF-κB) signaling pathway (Benedetti et al., 2014). Besides, a recent paper conducted by the Xiao’s group demonstrated that *Ureaplasma* spp. infection could suppress antimicrobial peptides expression, which was an important constituent of the innate immune system (Xiao et al., 2014). Nevertheless, these results favor the concept that mycoplasma species possess the ability to inhibit host immune responses and consequently ward off host immune surveillance and clearance, allowing for their survival within the host.

## Conclusion and Future Perspectives

Faced with the powerful and hostile host immune system, quite a few successful mycoplasma species still cause diseases and establish chronic, persistent infections. Therefore, over the past decades, insightful studies have been carried out to strengthen our understanding of the mechanisms evolved by mycoplasma to maintain their survival and interplay with host cells. In this review, we assembled and described several critical mechanisms by which mycoplasma subvert the host immune system to become long-term survivors.

In spite of the variety of strategies possessed by mycoplasma to escape immune damage, there is still a heavy burden to overcome. Above all, the mycoplasma genome coverage is limited, and completion is difficult and there are no effective and rapid means of genetic modification. Although the Tn4001 transposon or *oric*-plasmid has been used for single-gene knockouts, heavy screening work and low recombination frequency lead to appliance in rather few mycoplasma species (Karas et al., 2014; Li et al., 2015; Yamamoto et al., 2017). Therefore, we anticipate more advanced gene manipulation means will be developed in order to clarify unknown putative genes, which will be beneficial to better understand the cross-talk between mycoplasma and the host.

Innate immune function is the first line of the anti-infection immune system; however, there are few studies on whether other innate immune constituents are disturbed by mycoplasma, with the exception of what has been described in this review. In addition to bygone thioredoxin, Ohr, OsmC, peroxidoxin and even MrsA/B, additional protein enzymes have been recently identified to share the ability to resist ROS-mediated killing, but a single protein is of finite protective significance. Obviously, the capacity of mycoplasma to evade host immune killing is ultimately the result of multiple mechanisms working together. Therefore, to some degree, it is promising to identify new functional proteins to better understand the battle against oxidative stress damage.

Despite lacking a cell wall, some mycoplasma can produce capsular polysaccharides and/or exopolysaccharides (Neyrolles et al., 1998; Daubenspeck et al., 2009; Bertin et al., 2013, 2015; Simmons et al., 2013; Gaurivaud et al., 2016). Among these identified polysaccharides, some carbohydrate components have been characterized to have anti-inflammatory effects and contribute to biofilms formation (Totté et al., 2015). At the moment, few studies have been carried out regarding capsular polysaccharides and/or exopolysaccharides or their influence on the host during the process of mycoplasma infection, such as their participation in cell adhesion or anti-phagocytosis effects. Fortunately, mycoplasma species possess abundant lipid components for which we have an advanced understanding. Thus, the identification and analysis of some lipid constituent functions is also our target in the future.

Over the past 20 years, extensive studies have been carried out to fully understand the interaction between mycoplasma and pattern recognition receptors. It is still uncertain whether mycoplasma interferes with the signaling pathways mediated by pattern recognition receptors, modifies the receptors, or enhances the negative regulatory activities of some multifunctional adaptors or kinases, leading to a “brake” in the inflammatory response and thus regulating the intensity of the immune response and eventually facilitating persistent infection. More recently, *M. pneumoniae* has been shown to induce inflammatory responses through TLR4 and autophagy pathways (Shimizu et al., 2014). Although the ligand of TLR4 is LPS from other bacteria, it is unclear what specific components of the mycoplasma membrane could bind to TLR4.

In any case, cell invasion is the most direct evidence of mycoplasma evasion of the host immune system. Due to the lack of a stable cell invasion model and the low repeatability of results from different laboratories, the receptors and signaling pathways related to cell invasion are poorly understood. Taken together, with more precise studies, we will begin to gain a full understanding of this important pathogen which will inevitably initiate an upsurge in drug target and vaccine development studies.

## Author Contributions

LQ drafted the manuscript. YC modified the manuscript. XY conceived the idea.

## Conflict of Interest Statement

The authors declare that the research was conducted in the absence of any commercial or financial relationships that could be construed as a potential conflict of interest.
